# Therapeutic targets to reduce the contribution of pulmonary neutrophilic inflammation towards obesity-associated co-morbidities: a mini-review

**DOI:** 10.36811/ojpsr.2019.110006

**Published:** 2019-08-10

**Authors:** FN Claveles Casas, FS Barrera, CM Lopez, V Chacon I. del, MP Di Sciullo, DC Ramirez, SE Gomez Mejiba

**Affiliations:** 1 Laboratory of Experimental & Translational Medicine. IMIBIO-SL, CONICET-National University of San Luis, San Luis, 5700 San Luis, Argentina; 2 Laboratory of Experimental Therapeutics & Nutrition. IMIBIO-SL, CONICET-National University of San Luis, San Luis, 5700 San Luis, Argentina

**Keywords:** Obesity, Adipose tissue inflammation, Systemic inflammation, Neutrophil, Myeloperoxidase, Therapeutic target

## Abstract

Epidemiology and experimental models have shown a close link between adipose tissue inflammation, systemic inflammation and pulmonary neutrophilic inflammation, which predispose obese patients to pulmonary diseases, obesity-associated co-morbidities and cancer. Increased content and activation of neutrophils in the lung microvasculature, resulting from peripheral activation of neutrophils, and increased adhesion of neutrophils to the lung microvasculature are important factors explaining the increased susceptibility of obese patients towards respiratory diseases and loss of insulin sensitivity. Mechanism-based therapies to break this link are urgently needed to reduce pulmonary damage in obesity, due to the growing prevalence of obesity world-wide. Current research suggests that these approaches should be focused on, one or more of the following: reduction of macrophage activation at the adipose tissue, healthy growing of adipose tissue by induction of Nrf-2, inhibition of NF-κB activation, reduction of circulating neutrophil activation, blocking adhesins/selectins, inhibition of neutrophil activation by targeting NADPH oxidase-2 activation, inhibition of myeloperoxidase activity and scavenging of hypochlorous acid. These strategies are expected to reduce adipose tissue inflammation, peripheral inflammation, pulmonary neutrophilic inflammation and obesity-associated co-morbidities.

## Adipose Tissue and Systemic Inflammation in Obesity

Obesity is a frequent, costly and serious chronic inflammatory disease. This disease is characterized by a state of low-grade chronic inflammation resulting of a chronic positive energy-balance [1,2]. When adipose tissue capacity to store energy is overwhelmed, excess free fatty acids (FFAs) increase in circulation [3] where it can activate a number of vasculatures and circulating innate immune cells, such as monocytes and neutrophils [4,5]. Increased circulating activated neutrophils have been observed in obese patients [6]. This activation process may be caused by inflammatory factors such as end-oxidation products of macromolecules, as well as pro-inflammatory cytokines released by the inflamed adipose tissue [7,8]. Intestinal microbiota is also a factor to consider due to the increased intestinal permeability to endotoxin that can translocate into the systemic circulation and activate peripheral cells, as well as distant tissue vasculatures [1–9].

Adipose tissue inflammation is a multifactorial process that involves a number of causes, including adipose extracellular matrix stress, endotoxin translocation from the gut into circulation, macrophage infiltration, hypoxia, endoplasmic reticulum-stress and mitochondrial dysfunction [2,7,10,12]. Products released by the inflamed adipose tissue reach systemic circulation, where they can activate leukocytes and other tissue cells.

Adipose tissue macrophages derive from circulating monocytes. Under normal conditions these monocytes are differentiated into macrophages with an M2 anti-inflammatory phenotype; and their function is the patrol of the tissue for damaged/death cells, tissue reparation and remodeling [10]. However, under metabolic stress conditions caused by hypoxia, endoplasmic reticulum (ER)-stress and FFAs, monocytes are recruited in large numbers and differentiated into pro-inflammatory M1-macrophages [10,13]. The pro-inflammatory phenotype of these macrophages is caused mainly by activation of the nuclear factor-κB (NF-κB) signaling pathway [14]. This activation leads to expression of a number of pro-inflammatory cytokines/chemokines and large production of reactive biochemical species. Adipocyte hypertrophy and monocyte infiltration are the most relevant events in adipose tissue inflammation, because they are associated with the phenotypic switch of macrophage towards M1-proinflammatory cells located at hypoxic areas within the adipose tissue, crown-like structures [11,13,15].

**Figure 1:**
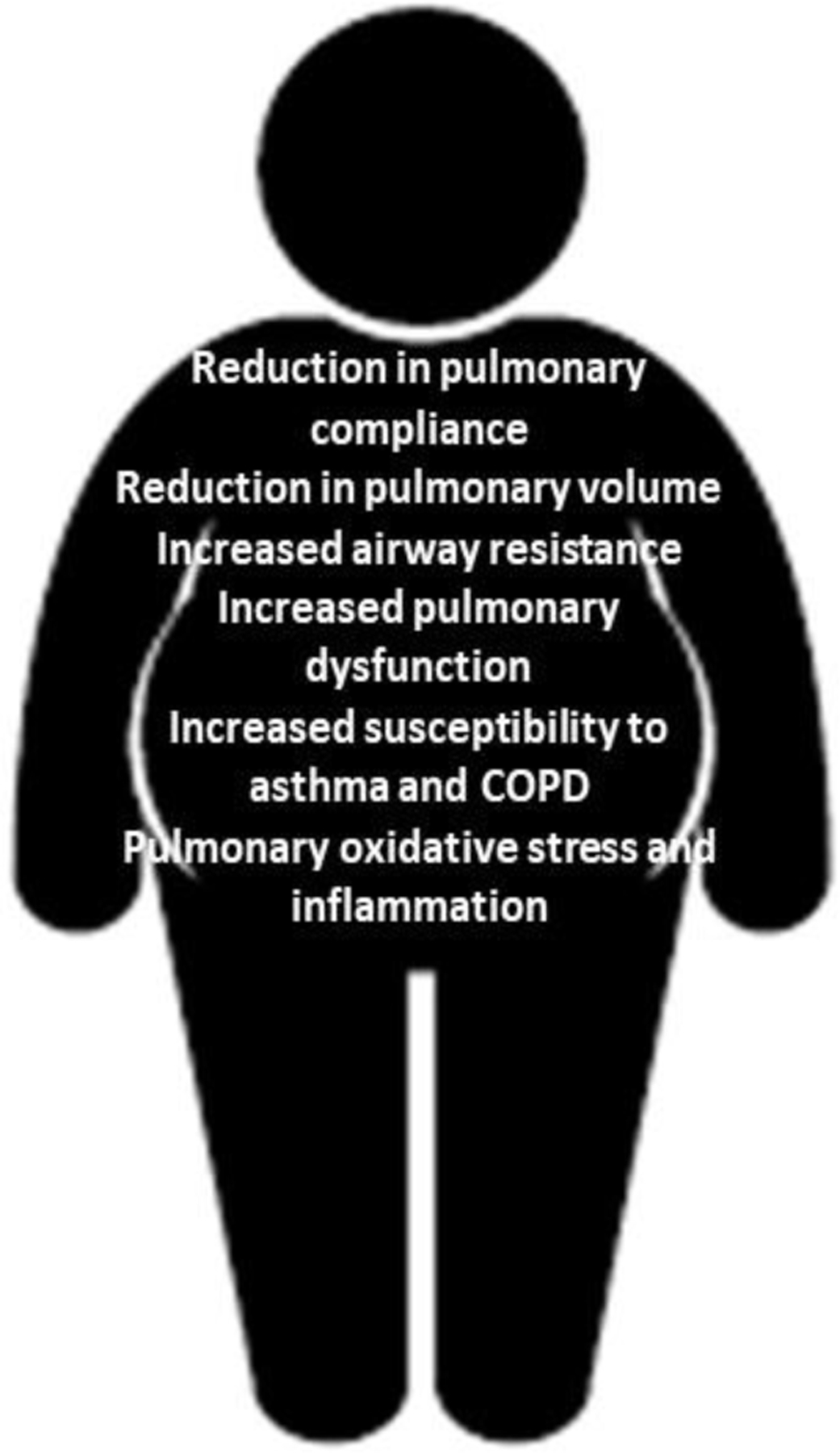
Pulmonary complications of obesity. Image shows a number of pulmonary complications observed in obese patients caused by adipose tissue-derived systemic inflammation. COPD, chronic-obstructive pulmonary disease.

Deregulated secretion of inflammatory mediators by a growing mass of fat tissue in obesity is a risk factor for biochemical stress in the lung of obese patients [16]. Figure 1 shows some of the pulmonary complications observed in obese patients and in animal models of obesity. These complications are the result of mechanical and biochemical pressures in obese patients and animals. Adipokines are proteins that are mainly or exclusively produced by adipocytes, and responsible for many of the pulmonary complications in obesity [17]. Examples include leptin, resistin and adiponectin, which have potential immunomodulatory effects in pulmonary cells [17,18]. Genetically obese *ob/ob* mice, which produce a mutated, non-functional form of leptin, show many of the same inflammatory changes as other models of obesity (including diet-induced obese mice), and *ob/ob* mice become both insulin resistant and diabetic [19]. These results indicate that leptin may not have a particularly important role in obesity-induced inflammation [20]. Adiponectin is considered to be an anti-inflammatory, insulin-sensitizing and cardioprotective protein [17,21–23]. It may cause these effects in several pathways; for example, by inducing anti-inflammatory cytokines such as IL-10 and IL-1Ra, through vascular mechanisms including enhancement of nitric oxide bioavailability, or by reducing endothelial cell–leukocyte adhesion [24,25].

In obesity, a low-grade chronic state of inflammation is responsible for multiple organs dysfunction [10]. Systemic inflammation produced by the inflamed fat has been correlated with the development of insulin resistance, a hallmark of type 2 diabetes mellitus (T2DM) as well as a number of chronic inflammatory diseases and cancer [2,26].

## The Lung Microvasculature Homes More Neutrophils in Obesity

**Figure 2:**
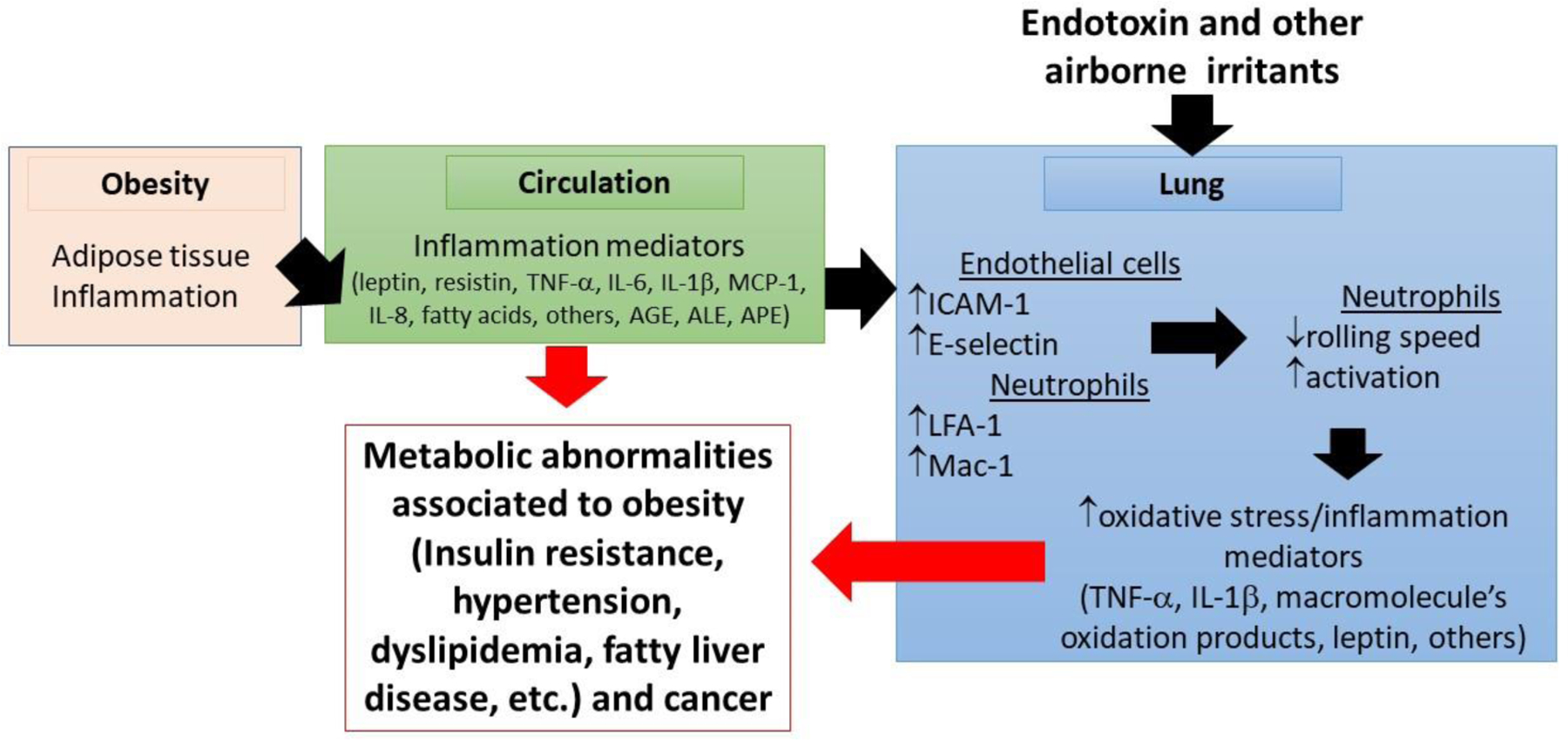
Obesity, pulmonary neutrophilic inflammation and obesity-associated metabolic abnormalities.

Inflammation mediators (pro-inflammatory cytokines/chemokines and macromolecule end-oxidation products) released by the adipose tissue [11] may activate both, circulating neutrophils and peripheral microvasculatures (Figure 2). Therapies aimed at reducing activation of neutrophils, recruitment of neutrophils into tissues and adhesion molecules in microvasculature may have clinical benefit in the future to overcome tissue damage induced by excessive leukocyte infiltration [6,27]. In addition, obese and leptin-supplemented mice are more sensitive to ozone-induced lung inflammation than their lean counterparts [28]. This evidence suggests that in obese patient’s leptin and/or resistin may cause lung inflammation by increasing neutrophil content, and possibly their activation, in the lung microvasculature [29–31]. In pathological conditions such as asthma and chronic obstructive pulmonary disease (COPD), it is likely that the tracheal-bronchial circulations are pivotal in the recruitment of circulating leukocytes [29,32]. Furthermore, the mechanism of the airway circulation for the recruitment of inflammatory cells in obesity after the inhalation of airborne pollutants requires further studies.

Adipose tissue inflammation in obesity produces a number of inflammation mediators. These cause activation of neutrophils in circulation and endothelial cells in the lung. This process results in increased content and activation of neutrophils and MPO-promoted oxidation reactions and pulmonary inflammation. Under this condition the lung acts as an extra source of inflammation mediators that contribute towards systemic inflammation and worsen obesity-associated co-morbidities and cancer. AGE, advanced-glycation end-products; ALE, Advanced-lipid peroxidation end-products; APE, Advanced-protein oxidation-end products.

Neutrophils are key sentinel cells of the innate immune system and are the premier cellular responders to acute inflammation [33,34]. For example, in models of acute lung injury ranging from acid aspiration to ischemia- reperfusion, depletion of neutrophils before the injury stimulus protects rabbits, rats and mice, respectively, from lung injury. These noninfectious models differ from the most common clinical causes of acute lung injury, pneumonia and sepsis, in which neutrophils are needed to control the infection. Nonetheless, animal work has demonstrated that the pro-inflammatory response of the neutrophil can lead to an increase in endothelial and epithelial permeability and, in the case of sepsis, shock and global organ injury [35,36].

The lung microcirculation has a concentration and traffic of neutrophils 30–100-times higher than any other large vasculatures in the human body, which might be further increased during obesity, making it a potential source of oxidants, oxidized products and pro-inflammatory cytokines upon exposure to air pollutants [37–40]. Airway inflammation requires an orchestrated series of molecular events whereby inflammatory cells leave the airway vasculature and migrate within the airway space [41]. Leukocytes are initially tethered to the endothelial cell surface of post-capillary venules, then roll, before firmly adhering and subsequently migrating out of the vasculature [42]. In obesity, circulating inflammation mediators derived from an inflamed adipose tissue, have been shown to produce oxidative stress in the brain and muscle tissue, both cardiac and skeletal, but the information in lung is limited [43]. Among the pro-inflammatory adipokines secreted in high quantities by the inflamed fat tissue, leptin, TNF-α, MCP-1, IL-1β and resistin have been shown to increase intercellular-adhesion molecule-1 (ICAM-1) expression in endothelial cells and to slow neutrophil rolling [16]. These adipokines and other inflammatory mediators in the serum of obese patients are thought to be a leading cause of systemic inflammation and thus obesity-associated metabolic disorders [37,40].

Leukocyte-endothelial cell interactions are mediated by various cell adhesion molecules [44]. These interactions are important for leukocyte extravasation and trafficking in all domestic animal species. An initial slowing of leukocytes on the vascular endothelium is mediated by selectins [45]. This event is followed by activation of β2-integrins after leukocyte exposure to cytokines and pro-inflammatory mediators, adherence of leukocyte β2 integrins to vascular endothelial ligands (*e.g.*, ICAM-1), extravasation of leukocytes into tissues through tight junctions of endothelial cells mediated by platelet and endothelial cell adhesion molecule-1 (PECAM-1), and perivascular migration through the extracellular matrix via β1 integrins [42,46]. Inhibiting excessive leukocyte egress and subsequent free radical-mediated damage caused by leukocyte activation may attenuate or eliminate tissue damage [47]. Several methods have been used to modify leukocyte infiltration in various animal models [48]. These methods include nonspecific inhibition of pro-inflammatory mediators and adhesion molecules by nonsteroidal anti-inflammatory drugs and glucocorticoids, inhibition of cytokines and cytokine receptors, and inhibition of specific types of cell adhesion molecules, with inhibitors such as peptides and antibodies to β2 integrins, and inhibitors of selectins, ICAMs, and vascular cell adhesion molecule-1 (VCAM-1) [46]. By understanding the cellular and molecular events in leukocyte-endothelial cell interactions, therapeutic strategies are being developed in several animal models and diseases in which neutrophilic inflammation plays a role.

## Oxidations Promoted by Pulmonary Neutrophilic Inflammation and Systemic Inflammation in Obesity

Previously an increased activation of neutrophils in the systemic inflammation induced by adipose tissue inflammation has been reported [49]. We have also shown increased of neutrophil accumulation and activation in the lung of diet-induced obesity mouse model [50]. Neutrophil activation was associated with more oxidative stress and inflammation in the lung and systemic environments [17,51,52]. This effect caused reduced insulin sensitivity that was improved by decreasing neutrophil homing, myeloperoxidase activation or by scavengers of HOCl (*unpublished data*). The main players in causing lung inflammation induced by environmental and metabolic stressors are neutrophils and myeloperoxidase (MPO), an enzyme contained in neutrophils’ azurophilic granules [51,53]. MPO is the only mammalian enzyme that, under physiological conditions, produces HOCl [54]. MPO can produce a number of hypohalous (e.g., HOCl, IOH and BrOH) and pseudo-hypohalous (e.g., SCNOH) compounds [55]. MPO uses H_2_O_2_ to oxidize chloride anions into hypochlorous acid/hypochlorite (HOCl/^−^OCl, from now on HOCl), a potent oxidizing agent [56]. Hypochlorous acid further reacts with other biological molecules to generate secondary reactive species, such as chloramines and NO_2_Cl [56,57]. Determination of blood MPO and HOCl-modified proteins have been suggested a predictive biomarker of cardiovascular accident [52].

Activation of neutrophils and production of HOCl in the lung vasculature causes oxidative stress and inflammation in the lung [50,52]. On the other hand, inhalation of air pollutants is associated with altered serum biomarkers of oxidative stress and inflammation (BOSI) and a higher incidence of insulin resistance in swine farm workers [58]. The incidence of cardiovascular diseases has been reported to increase in humans driving cloudy routes during rush hours [59,60]. The prevalence of airway sensitization in obese is higher than lean subjects [51,61] (See Figure 1). This evidence has been also supported in obese experimental animals [32]. Therefore, it is possible that inhalation of air pollutants increases the incidence of systemic inflammation and obesity-associated metabolic complications in obese patients (BMI>30 Kg/m^2^) in relation to normal weight subjects [2]. This disparity between obese and lean patients to develop obesity-associated diseases upon inhalation of air pollutants can be prevented by blocking free radical processes in the obese lung with specific antioxidants that target MPO or MPO-derived oxidants, such as HOCl (Figure 2). Using genetically modified mice which are unable to release superoxide anions and superoxide-derived oxidants from NAD(P)H oxidases (NOX), it has been found that PM2.5 increases obesity and insulin resistance *in vivo* by NAD(P)H oxidase-derived superoxide anions [62]. Superoxide anions and their derived oxidants including hydrogen peroxide and peroxynitrite are known to suppress vascular endothelial function and lower insulin responsiveness [62]. The NAD(P)H oxidase, a membrane-bound enzyme complex, consists of multiple subunits including p22^*phox*^, p40^*phox*^, p47^*phox*^, p67^*phox*^, NoxO1, NoxA1, Rac1, andgp91^*phox*^-related unique isoforms of Nox [63]. The complex is normally latent in neutrophils and is activated to assemble in the membranes during respiratory burst [64]. The choice of Xu et. al. to target the p47^*phox*^ subunit of NAD(P)H was based on its homology in both phagocytic and nonphagocytic inflammatory pathways that are implicated in NAD(P)H-mediated insulin resistance [65]. In an elegant series of experiments using C57BL6 and p47^*phox*^ homozygous knockout mice, the investigators have systematically assembled evidence that PM2.5 exposure when the animals were 3-week-old young pups, induced a later life phenotype that manifested increase in abdominal fat, increased adipocyte size, and heightened inflammatory cellular response [65]. This study suggests that early life exposure to PM2.5 scale particulate pollution primes the system toward developing insulin resistance and a proinflammatory vascular phenotype in later life, likely via NAD(P)H oxidase–derived superoxide anions.

## Therapeutic Targets

The link between adipose tissue inflammation, systemic inflammation and pulmonary neutrophilic inflammation should be interesting to break because pulmonary inflammation can contribute to the systemic pool of inflammation mediators that can worsen systemic inflammation, and consequently obesity-associated co-morbidities, such as insulin resistance (Figure 2). Obviously, the most desired therapeutic approach is that that can control appetite or increase energy expenditure avoiding the chronic positive energetic balance [66]. On another hand, change or transplant of microbiota may be a chance to revert changes in gut microflora in obese patients [67]. However, this option has been recently questioned after the US Food and Drug Administration informed the death of one patient by contamination with *Clostridium difficile* during a clinical trial. Consequently, more research is needed on this target.

Adipose tissue inflammation is a consequence of a metabolic inflammation process [10]. Macrophage activation by either FFAs or endotoxin should be targeted. The common link between these two stressors is the NF-kB signaling pathway [14], Table 1. Indeed, endotoxin and FFA (palmitic-, miristic- and stearic- fatty acids) activate innate immune cells by binding to TLR-4 and TLR-2, respectively, thus contribute to metabolic inflammation in the adipose tissue. Activation of TLRs in macrophages leads to signal transduction that induces NF-κB activation-the master regulator of the inflammatory response [14]. Tissue cell activation with production of chemokines may be anther target, because these attract monocytes that differentiate into M1 macrophages. MCP-1 and 4-1BB are interesting target and the use of peptidomimetics should be further explored to interfere in the binding to its receptors in monocytes [10.^68^,^69^] The microenvironment of metabolically irritated adipose tissue by FFAs may be another target. In the irritated and hypoxic environment of metabolically stressed adipose tissue, recruited monocytes differentiate into M1-inflammatory macrophages [12], which produce large quantities of pro-inflammatory cytokines and reactive biochemical species (reactive oxygen species, nitrogen reactive species, and macromolecule end-oxidation products) [10]. Thus, the first level of intervention should be improving the storage of FFAs [70,71], See Table 1.

Previously has been shown that Nrf2 is an important transcription factor controlling the expression of two transcription factors master regulators of adipogénesis [70]. These transcription factors are PPAR-γ and CEBP-α [72]. These transcription factors control the expression of adipogenic enzymes that catalyze the synthesis of tryglicerides—the safest way to store excess FFAs [73]. Thus, by activating Nrf-2 circulating concentrations of FFAs can be reduced and systemic inflammation reduced as well as metabolic complications of obesity, such as pulmonary inflammation [71]. In this regard we have found that the nitrone spin trap DMPO can block alternative activation of macrophages by the endotoxin lipopolysaccharide [74–76]. This spin trap also causes transcriptomic changes related to an inhibition of the expression of genes related to inflammatory activation [74]. The inhibitory effect of DMPO on LPS-induced macrophage activation was due to the direct binding of the spin trap to four specific residues in the TIR domain of TLRs (TLR2) named BB-loop [75].

Myeloperoxidase and HOCl may be another target for preventing pulmonary neutrophilic inflammation in obesity (Table 1). Indeed, neutrophils, MPO and markers of HOCl-promoted oxidations have been observed in the lung of obese patients and in mouse models of diet-induced obesity and metabolic syndrome. Thus, the development of inhibitors of MPO or scavengers of HOCl may prove to be effective in preventing adipose tissue dysfunction (adipokine dysbalance, insulin resistance and cell death). Targeting the lung by using MPO inhibitors or HOCl scavengers may prove to be effective to prevent the contribution of pulmonary neutrophilic inflammation towards systemic inflammation, the leading cause of obesity-associated metabolic abnormalities (Figure 2). Thus, targeting inflammation and oxidative stress in the growing adipose tissue and lung is an attractive strategy to prevent obesity-associated co-morbidities and probably obesity associated cancers.

## Concluding Remarks

Obesity is state of chronic low-grade inflammation caused by a chronic positive energy balance. This causes adipose tissue inflammation with infiltration of monocytes that acquire an inflammatory phenotype. The adipose tissue is inflamed and oxidants overwhelm the antioxidant capacity causing oxidative stress. Oxidized products as well as pro-inflammatory cytokines are secreted into the systemic circulation where it can activate neutrophils as well as activate NF-κB, and other inflammatory transcription factors leading to expression of neutrophil adhesion molecules in the lung microvasculature. Oxidative stress and inflammation in the lung increased the systemic pool of pro-inflammatory and end-oxidation products that reduce insulin sensitivity and cause other metabolic abnormalities-associated to obesity. Therapeutic strategies to reduce the pulmonary contribution towards systemic inflammation are needed. These should be focused in reducing neutrophilic inflammation in the lung microvasculature (see Table 1). They include improving fatty acid storage in adipose tissue by inducing Nrf-2, reducing inflammatory activation of adipose tissue macrophages, inhibiting MPO activity and scavenging HOCl in the lung. These approaches are active fields of research in our laboratories.

## Figures and Tables

**Table 1:** Targets and therapeutic opportunities to reduce obesity-associated pulmonary neutrophilic inflammation and its contributions towards obesity co-morbidities*

Target	Pharmacological strategy	Expected effects
Circulating free fatty acids	Increase Nrf2 pathway activation	▪ Reduction of pro-inflammatory free fatty acids in circulation ▪ Detoxification of electrophils and products of lipid peroxidation ▪ Increments in synthesis of antioxidants
Neutrophil homing in the lung	Reduce homing of neutrophils in microvasculature (e.g. reduce NF-κB activation with DMPO, peptides, etc.)	▪ Reduction of NF-κB activation ▪ Inhibition of the expression of adhesion molecules in the microvasculature ▪ Inhibition of the phenotypic switch of macrophages towards an M1 phenotype
Systemic inflammation	Anti-pro-inflammatory cytokines and chemokines antibodies (TNF-α, IL-1a, IL-6, IL-8, MCP-1, etc.)	▪ Reduction of the availability of inflammation mediators in circulation
Pulmonary-netrophil activation	NOX-2 inhibitors (peptides, apocynin, etc.)	▪ Reduction of neutrophil activation and release of MPO
PulmonaryMPO activity	Inhibitors of MPO chlorinating activity (ABAH, natural products, peptides, etc.)	▪ Reduction of HOCI production
HOCI concentration	Scavenging of HOCI to reduce damage to macromolecules (resveratrol, taurine, natural products, etc.)	▪ Scavenge of HOCI before it reacts with biological targets
Damaged macromolecules in the lung	Antioxidants to protect and repair damaged macromolecules (e.g., spin traps, L-ascorbate, GSH)	▪ Reduction of the pro-inflammatory effects of end-oxidation products

* See text for references.
